# Sex-Specific Differences in Motor-Unit Remodeling in a Mouse Model of ALS

**DOI:** 10.1523/ENEURO.0388-19.2020

**Published:** 2020-02-20

**Authors:** Éric Martineau, Adriana Di Polo, Christine Vande Velde, Richard Robitaille

**Affiliations:** 1 Département de neurosciences, Université de Montréal, Montréal, Québec H3C 3J7, Canada; 2 Centre de Recherche du Centre Hospitalier Universitaire Sainte-Justine, Montréal, Québec H3T 1C5, Canada; 3 Centre de Recherche du Centre Hospitalier de l’Université de Montréal, Montréal, Québec H2X 0A9, Canada; 4 Groupe de Recherche sur le Système Nerveux Central, Université de Montréal, Montréal, Québec, H3C 1J7, Canada

**Keywords:** amyotrophic lateral sclerosis, motor-unit, neuromuscular junction, reinnervation, sex-specific differences, superoxide dismutase

## Abstract

Progressive loss of neuromuscular junctions (NMJs) is an early event in amyotrophic lateral sclerosis (ALS), preceding the global degeneration of motor axons and being accompanied by new axonal sprouting within the same axonal arbor. Some aspects of ALS onset and progression seem to be affected by sex in animal models of the disease. However, whether there are sex-specific differences in the pattern or time course of NMJ loss and repair within single motor axons remains unknown. We performed further analysis of a previously published *in vivo* dataset, obtained from male and female SOD1^G37R^ mice. We found that NMJ losses are as frequent in male and female motor axons but, intriguingly, axonal sprouting is more frequent in female than male mice, resulting in a net increase of axonal arborization. Interestingly, these numerous new axonal branches in female mice are associated with a slightly faster decline in grip strength, increased NMJ denervation, and reduced α-motor neuron survival. Collectively, these results suggest that excessive axonal sprouting and motor-unit (MU) expansion in female SOD1^G37R^ mice are maladaptive during ALS progression.

## Significance Statement

Sex-specific differences in amyotrophic lateral sclerosis (ALS) progression have been identified in patients and in some animal models of the disease. However, the physio-pathologic changes underlying these disparities remain poorly defined. In this study, we identified that the pattern of motor axon retraction and regrowth in skeletal muscles is a novel factor to consider in our understanding of sex-linked differences in ALS. Analysis of single motor axons in a model of ALS identified that female motor axons were more likely to form compensatory branches, which was associated with a worse phenotype. These surprising findings highlight the necessity to more systematically evaluate the prevalence of sex-specific differences across animal models of ALS and in patients.

## Introduction

Amyotrophic lateral sclerosis (ALS) is a neurodegenerative disease affecting motor neurons. The early dysfunction and loss of neuromuscular junctions (NMJs) is a key event in patients and animal models of the disease (Fischer et al., 2004; Pun et al., 2006; Armstrong and Drapeau, 2013a,b; Clark et al., 2016; Tallon et al., 2016; Tremblay et al., 2017; Chand et al., 2018). A recent study has shown in SOD1^G37R^ mice that NMJ loss within single motor-units (MUs), defined as a motor neuron and the muscle fibers it innervates, is initially slow and asynchronous (Martineau et al., 2018). These losses are accompanied by the formation of new collateral axonal branches (expansions), reinnervating other nearby NMJs (Martineau et al., 2018). Importantly, this period of dynamism precedes global axonal degeneration by several weeks, revealing that NMJ loss is initially a local process involving constant axonal retraction and growth.

Sex-specific differences in disease onset and progression have been observed in patients and in some animal models of ALS (McCombe and Henderson, 2010). In humans, ALS onset tends to be delayed in women with most studies reporting a delay between 0.9 and 5.5 years in the mean or median age at diagnosis (McCombe and Henderson, 2010; Chen et al., 2015). This tendency is further reflected in the sex ratio for the incidence of ALS. Indeed, while the overall male-to-female ratio is around 1.3, the ratio is 3.98:1 for onsets occurring before 49 years of age and gradually equalizes with age (Manjaly et al., 2010; McCombe and Henderson, 2010). Similarly, disease onset is slightly delayed in SOD1^G93A^ female mice compared with male mice (Pfohl et al., 2015). However, whether sex affects neuromuscular dysfunction and NMJ loss in ALS remains ill-defined. Previous studies using electromyographic recordings did not identify sex-specific differences in the global time course and pattern of motor axon loss in SOD1^G93A^ mice (Hegedus et al., 2009) or in ALS patients (McComas et al., 1971; Dengler et al., 1990; Dantes and McComas, 1991; Schmied et al., 1999). Interestingly though, a recent study identified that women with ALS were twice as likely of having antibodies against two NMJ components, agrin and LRP4 (Rivner et al., 2017). Furthermore, Moloney et al. (2017) identified sex-specific differences in botulinum toxin-induced axonal sprouting in mice, with female mice notably exhibiting more sprouting from ALS-vulnerable fast-fatigable MUs. These recent findings raise the possibility that sex-specific differences may impact local NMJ dynamism in ALS, which could be an important factor to consider for therapeutic development.

To address this unexplored question, we examined whether NMJ denervation and reinnervation within single motor axons occur similarly between male and female SOD1^G37R^ mice. We found that motor axons were more likely to form new axonal branches in female than in male SOD1^G37R^ mice. Interestingly, these compensatory expansions were associated with increased neuronal loss and NMJ denervation in female mice, suggesting that they may be detrimental to disease progression.

## Materials and Methods

### Animals

SOD1^G37R^/YFP (*SOD1^+/-^*; YFP^+/-^) transgenic mice were previously described (Martineau et al., 2018). Briefly, they were obtained by breeding male lox*SOD1^G37R^* mice (RRID:IMSR_JAX:016149) with heterozygote female *Thy1*-YFP, line H, mice (B6.Cg-Tg(Thy1-YFP)HJrs/J; The Jackson Laboratory, stock number 003782; RRID:IMSR_JAX:003782). *Thy1*-YFP mice from line H were used due to their sparse expression of the yellow-fluorescent protein (YFP) in lower motor neurons (Feng et al., 2000) allowing visualization of single motor axons. Parent lines were maintained on a C57BL6/J background. Importantly, transmission of both transgenes follows a Mendelian autosomal pattern of inheritance, ruling out the possibility that either transgene is integrated on a sex chromosome. Disease progression and motor function were monitored via weekly weight and grip strength measurements (BioSeb, BIO-GS3). Animals were sacrificed using a lethal dose of isoflurane. All experiments were performed in accordance with the guidelines of the Canadian Council on Animal Care, the Comité de Déontologie sur l’Expérimentation Animale of Université de Montréal (protocol #18–040), and the CRCHUM Institutional Committee for the Protection of Animals (protocol #N16008CVV and #N15047ADPs).

### Repeated *in vivo* imaging and image analysis

Procedures for the repeated *in vivo* imaging of the *Tibialis anterior* muscle and image analysis were described in the original publication of the *in vivo* imaging dataset (Martineau et al., 2018). Briefly, mice were imaged every two weeks, for five to six sessions (10–12 weeks). Mice were anesthetized with 2–3% isoflurane and the *Tibialis anterior* muscle was exposed by making a rostro-caudal incision on the exterior face of the hindlimb. Postsynaptic nAChRs were labeled by applying a non-blocking concentration of Alexa Fluor 594-conjugated α-bungarotoxin (BTX; 5 μg/ml, in sterile lactated Ringer for 10 min; Molecular Probes, Fisher Scientific) on the first session. BTX was reapplied only when the labeling was too dim, on session 5 or 6. Images were obtained using a Neo-sCMOS camera (Andor) mounted on an upright Optiphot-2 microscope (Nikon) equipped with a 20× water immersion objective (0.4 NA, Nikon). Excitation light and fluorescence emission were filtered using a Brightline Pinkel filter set optimized for CFP/YFP/HcRed (CFP/YFP/HcRed-3×-A-000; Semrock). The wound was then closed using 5–0 and 6–0 vicryl sutures and tissue glue (GLUture; Abbott Laboratories, WPI). Mice were administered Buprenorphine (3 μg/10 g body weight; every 8 h, three doses; Temgesic, CEVA Animal Health Ltd) by subcutaneous injections. All SOD1 animals with YFP-positive surface motor axons which could be reliably followed for at least three sessions and with at least three NMJs near the surface were included in this dataset. The vast majority of imaged motor axons belong to fast-fatigable MUs as previously reported for this dataset (Martineau et al., 2018).

### Whole-mount NMJ immunolabeling and motor neuron counts

Procedures for the whole-mount muscle and spinal cord immunostaining were previously described in detail (Martineau et al., 2018). Images were acquired on a Zeiss LSM 880 confocal microscope with a 20× water immersion objective (N.A. 1.0). No image manipulations were performed after acquisition, except for linear contrast adjustments for figure presentation. Motor neurons in both ventral horns were counted from 15 to 20 sections per animal, separated by at least 90 μm. The Allan Brain Atlas Mouse Spinal cord reference set was used to ensure that all analyzed sections were in the lumbar spinal cord. All ChAT-positive cells in the ventral horn were considered as motor neurons. NeuN-positive motor neurons were counted as α-motor neurons while NeuN-negative motor neurons were counted as γ-motor neurons (Shneider et al., 2009; Lalancette-Hebert et al., 2016).

### Statistical analysis

**Figure 1.**
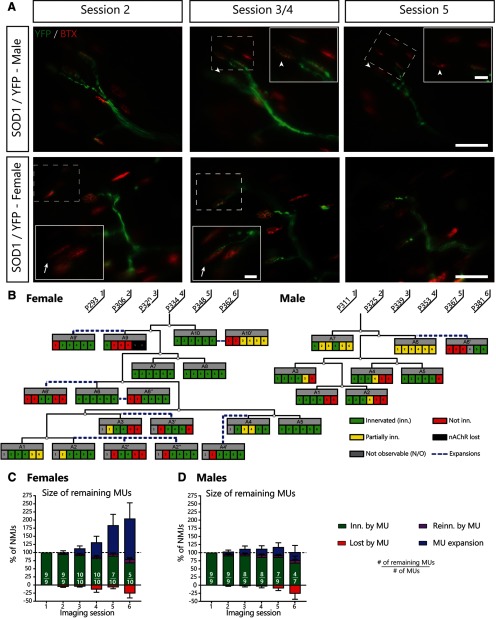
Sex-specific differences in MU dynamics in SOD1^G37R^/YFP mice. ***A***, Examples of MU arbors from a male and a female SOD1/YFP mice during three sessions (male: session 2, 3, and 5; female: session 2, 4, and 5), with higher magnification on-focus insets of NMJs of interest (digital zoom, dashed box in low magnification). Green: YFP-labeled axon; red: nAChR. Note how the imaged motor axon retracted from two NMJs (arrowheads) between sessions 3 and 5 in the male example. Furthermore, note the formation of a new axonal branch between sessions 2 and 4 in the female example (arrow), innervating an NMJ that previously did not belong to this MU (MU expansion). ***B***, MU dynamic diagram of the MUs shown in ***A***, showing that MU are much more likely to expand in female SOD1^G37R^/YFP mice. Black lines represent the axonal arborization and each box represents a single NMJ. Note how asynchronous NMJ losses occurred in both these MUs, but how partial losses seem more frequent in the male MU. ***C***, ***D***, Histograms showing the average proportion of NMJs from the initial pool which are innervated (green), re-innervated (purple), or lost (red) by the MU and the proportion which are gained (blue) for female (***C***) and male (***D***) SOD1^G37R^/YFP mice. Numbers in the histogram bars represent the number of remaining MU arbors (those which did not globally degenerate) over the total number of MUs observed. Again, note how MU expansions are more frequent in female than in male mice. Scale bar: 100 μm (low magnification) and 25 μm (high magnification).

Comparison of the frequency of expansions and asynchronous losses between male and female SOD1^G37R^ mice was performed by using a multivariate Generalized linear model (GLM) with Poisson’s or logistic distribution (Fig. 1). The cumulative number of expansions or the cumulative number of asynchronous losses over the total number of NMJs innervated by that MU at any time points for each animal were used as the dependent variables. A logistic distribution was used for asynchronous losses as they represent a number of events over a define number of trials (number of imaged NMJs innervated by that MU). Poisson’s distribution was used for the expansions as they represent events which occur over a period of observation (as opposed to a define number of trials). For this analysis, the sample size (number of MU arbors) and the number of biological replicates (number of animals) are indicated in the text; *p* values smaller than 0.05 (α = 5%) were considered statistically significant. Analyses were performed in the SPSS 24.0.0.0 (IBM) software.

**Figure 2.**
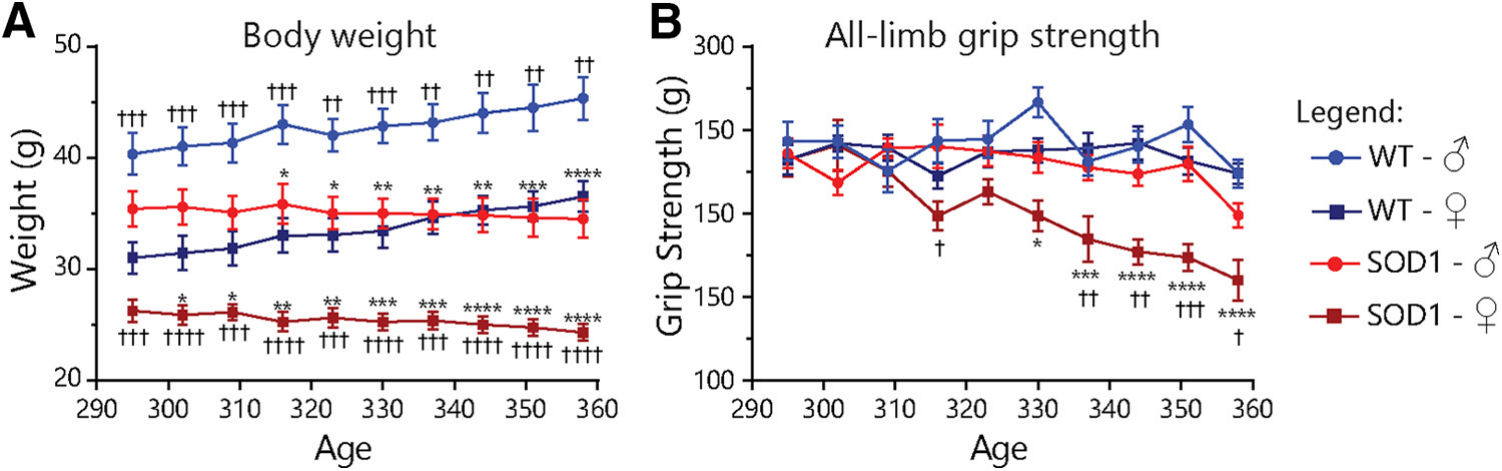
Motor function declines slightly faster in female SOD1^G37R^/YFP mice. ***A***, ***B***, Body weight (***A***) and all-limb grip strength (***B***) curves of male and female (light and dark colors, respectively) WT/YFP and SOD1^G37R^/YFP mice (blue and red, respectively) during the symptomatic stages (WT female: *N* = 14; WT male: *N* = 6; SOD1 female: *N* = 8; SOD1 male: *N* = 12). Data are presented as mean ± SEM. Main effects and interactions are reported in Table 1 and in the text. Asterisks and crosses represent biologically relevant statistically significant differences in the *post hoc* test (Tukey’s multiple comparisons). Asterisks represent differences compared with the other genotype, while crosses represent differences compared with the other sex; */†*p* < 0.05, **/††*p* < 0.01, ***/†††*p* < 0.001, ****/††††*p* < 0.0001.

**Figure 3.**
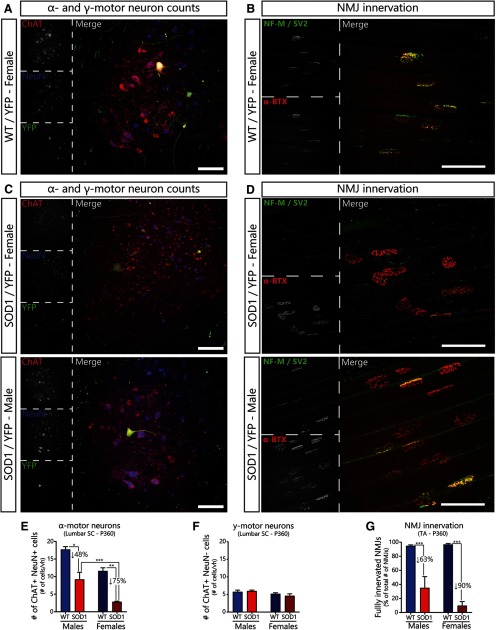
α-motor neuron loss and NMJ denervation are slightly more pronounced in female SOD1^G37R^/YFP mice at P360. ***A***, Representative image of lumbar spinal cord sections from a female WT/YFP mice at P360 (red: ChAT; blue: NeuN; green: YFP). ***B***, Representative image of NMJs in the *tibialis anterior* muscle of a female WT/YFP mice at P360 (red: α-BTX; green: NF-M and SV2). ***C***, Representative examples of lumbar spinal cord sections from female (top) and male (bottom) SOD1^G37R^/YFP mice at P360. ***D***, Representative images of NMJs in the tibialis anterior muscle of female (top) and male (bottom) SOD1^G37R^/YFP mice at P360. ***E***, ***F***, Quantification of the number of α-motor neurons (***E***, ChAT+ NeuN+ cells) and γ-motor neurons (***F***, ChAT+ NeuN– cells) per ventral horn at P360 (WT female: *N* = 4; WT male: *N* = 2; SOD1 female: *N* = 4; SOD1 male: *N* = 5). ***G***, Quantification of the percentage of fully innervated NMJs in the tibialis anterior at P360 (WT female: *N* = 4, *n* = 1078; WT male: *N* = 2, *n* = 446; SOD1 female: *N* = 4, *n* = 977; SOD1 male: *N* = 5, *n* = 820). Data are presented as mean ± SEM. Main effects and interactions are reported in Table 2 and in the text. Asterisks represent biologically relevant statistically significant differences in the *post hoc* test (***E***, ***F***, Tukey’s multiple comparisons; ***G***, Holm–Sidak’s correction); *p < 0.05, **p < 0.01, ***p < 0.001. Scale bar: 100 μm.

When the effect of two or three independent variables were compared (Figs. 2, 3), two-way or three-way ANOVAs were used, with (for motor behavior and weight) or without repeated-measures (RM; for motor neuron counts). For the *post hoc* test, Tukey’s multiple comparisons was used (referred to as “ANOVA *post hoc* test”). For NMJ innervation (Fig. 3), the main effects of each variable were compared using a GLM with a logistic distribution and Holm–Sidak’s correction was applied to all pairwise comparisons for the *post hoc* test (referred to as “GLM *post hoc* test”) as previously described (Tremblay et al., 2017). Data are presented as mean ± SEM in the histograms and in the text. *N* represents the number of biological replicates (animals), while *n* represents the number of observations (number of NMJs unless otherwise stated); *p* values smaller than 0.05 (α = 5%) were considered statistically significant. These analyses were performed in the GraphPad Prism 7.0 software, with the exception of the analysis for the NMJ innervation and the three-way ANOVAs with RM which were made in SPSS 24.0.0.0. For the purpose of clarity, only essential main effects and *post hoc* comparisons are presented in the text. All other comparisons are reported in Tables 1, 2 (main effects) or are represented in the graphs (*post hoc* comparisons).

**Table 1:** Effect of time, sex, and genotype on disease progression in SOD1^G37R^ mice

Analysis	Statistical test	Main effect	*p* value
Body weight (Fig. 2*A*)	RM three-way ANOVA	Time	<0.001***
		Genotype	<0.001***
		Sex	<0.001***
		Time × genotype	<0.001***
		Time × sex	0.854
		Sex × genotype	0.868
		Time × sex × genotype	0.397
Grip strength (Fig. 2*B*)	RM three-way ANOVA	Time	<0.001***
		Genotype	<0.001***
		Sex	<0.001***
		Time × genotype	<0.001***
		Time × sex	0.007**
		Sex × genotype	0.023*
		Time × sex × genotype	0.011*

Bold values represent statistically significant differences (*p* < 0.05).

**Table 2:** Effect of sex and genotype on neuronal survival and NMJ innervation in SOD1^G37R^ mice

Analysis	Statistical Test	Main effect	*p* value
α-Motor neurons counts (Fig. 3*E*)	Two-way ANOVA	Genotype	<0.001***
		Sex	0.002**
		Sex × genotype	0.941
γ-Motor neurons counts (Fig. 3*F*)	Two-way ANOVA	Genotype	0.673
		Sex	0.042*
		Sex × genotype	0.365
NMJ innervation (Fig. 3*G*)	GLM, logistic distribution	Genotype	<0.001***
		Sex	0.431
		Sex × genotype	0.019*

Bold values represent statistically significant differences (*p* < 0.05).

## Results

To investigate sex-linked differences in NMJ loss in ALS, we first asked whether NMJ denervation and axonal sprouting occurred similarly in male and female SOD1^G37R^. We previously reported that single motor axons in SOD1^G37R^ mice asynchronously retracted from some NMJs over time (a process referred to as asynchronous NMJ losses), while also forming new branches to innervate nearby postsynaptic sites (expansions; Martineau et al., 2018). Here, we took advantage of this open-access dataset and performed a secondary analysis comparing motor axon dynamics between male and female SOD1^G37R^ mice.

This dataset was obtained by repeatedly imaging single MUs and their NMJs in the *tibialis anterior* during disease progression every two weeks for five to six sessions (56–76 d). Single motor axons were visualized by using SOD1^G37R^ mice expressing the YFP in a subset of motor neurons (SOD1^G37R^/YFP-H mice) and postsynaptic nicotinic acetylcholine receptors were labeled with Alexa Fluor 594-conjugated BTX. A total of 253 NMJs, belonging to 19 different MU arbors (males: 102 NMJs, 9 MU arbors, seven animals; females: 151 NMJs, 10 MU arbors, four animals) were followed. NMJ losses or axonal sprouting innervating new NMJs (expansions) were recorded over time.

### MU expansions are more frequent in females SOD1^G37R^ mice

Interestingly, we observed that MU dynamics were vastly different between males and females (Fig. 1*A*,*B*). While a similar number of axonal branches retracted from postsynaptic sites (complete asynchronous NMJ losses) in male and female mice over all imaging sessions [Fig. 1*C*,*D*; males: 12/102 NMJs (12%) vs females: 26/151 NMJs (17%); GLM; *p *=* *0.592], female MUs were strikingly more likely to form expansions [Fig. 1*C*,*D*; males: 14/102 NMJs (14%) vs females: 62/151 NMJs (41%); GLM; *p *=* *0.004]. This difference was so pronounced that only female MUs exhibited a net increase in size, while expansions barely masked the losses in male MUs (Fig. 1*C*,*D*; average MU size on session 6, males: 99 ± 31% vs females: 204 ± 58%). Moreover, male MUs exhibited slightly more partial losses (partial retraction of the motor axon) than female MUs (males: 18/102 NMJs (18%) vs females: 12/151 NMJs (8%); GLM; *p *=* *0.029). Importantly, these differences are not due to differences in observation times or in the initial size of axonal arbors between sexes, i.e., more observations due to having observed one group for a longer period of time or having imaged larger arbors, as these parameters were comparable between groups (females MUs: 58 sessions, 88 initial NMJs vs males MUs: 48 sessions, 88 initial NMJs).

### Grip strength, but not body weight, declines slightly faster in female SOD1^G37R^ mice

To investigate whether these differences in MU dynamics were associated with differences in disease progression in this mouse line, we analyzed the disease course of male and female SOD1^G37R^ mice during the symptomatic phase by using two well-described and reliable methods: all-limb grip strength and body weight (Fig. 2; Table 1; Lobsiger et al., 2009, 2013; Mesci et al., 2015). Male and female SOD1^G37R^/YFP mice exhibited a parallel, age-dependent, decline in body weight relative to sex-matched WT/YFP animals (Fig. 2*A*; Table 1; RM three-way ANOVA; interaction between time and genotype: *p *<* *0.001; interaction between time, genotype and sex: *p *=* *0.397), as previously reported (Lobsiger et al., 2013). Surprisingly though, decline in all-limb grip strength was observable approximately a month earlier in female than in male SOD1^G37R^/YFP mice (Fig. 2*B*; Table 1; RM three-way ANOVA; interaction between time, genotype and sex: *p *=* *0.011). Hence, motor deficits occur earlier in female SOD1^G37R^/YFP mice compared with male mice.

### Motor neuron loss and NMJ denervation are exacerbated in female SOD1^G37R^ mice

To further validate this tendency, NMJ innervation in the *Tibialis anterior* and α- and γ-motor neuron survival in the lumbar spinal cord of male and female SOD1^G37R^/YFP mice at P360 were analyzed (Fig. 3; Table 2). Consistent with the *in vivo* results, α-motor neuron degeneration and NMJ denervation were already severe at P360 in both male and female SOD1^G37R^ mice (respectively, Fig. 3*A*,*C*,*E*; two-way ANOVA; effect of genotype: *p *=* *0.0002 and Fig. 3*B*,*D*,*G*; GLM; effect of genotype: *p *<* *0.001). Importantly, survival of γ-motor neurons was unchanged in SOD1^G37R^/YFP mice compared with WT/YFP mice, despite females having slightly less γ-motor neurons in general (Fig. 3*F*; Table 2; two-way ANOVA; effect of genotype: *p *=* *0.673; effect of sex: *p *=* *0.0420).

Consistent with the grip strength results, we found that α-motor neuron degeneration was increased in female SOD1^G37R^/YFP mice compared with male SOD1^G37R^/YFP mice (Fig. 3*E*; SOD1 males, 48% reduction vs SOD1 females, 75% reduction; ANOVA *post hoc* test; *p *=* *0.0304), although the global interaction term was not significant (Table 2; two-way ANOVA; interaction: *p *=* *0.9410). Similarly, loss of NMJ innervation in SOD1^G37R^/YFP mice tended to be greater in females than in males (Table 2; GLM, interaction; *p *=* *0.019), although no specific differences were observed between male and female SOD1^G37R^/YFP mice (Fig. 3*G*; SOD1 males vs SOD1 females; GLM *post hoc* test; *p *=* *0.121). Interestingly, NMJ innervation and α-motor neuron counts were more variable in SOD1^G37R^/YFP males than in SOD1^G37R^/YFP females (Fig. 3*E*,*G*), further suggesting that some individual male mice may have been less affected at that time point. In sum, these results confirm that α-motor neuron degeneration seems to be more advanced in female than in male SOD1^G37R^/YFP mice at P360.

## Discussion

Our results show that female fast-fatigable MUs (surface of the tibialis anterior; see Materials and Methods) are much more likely to form expansions in SOD1^G37R^/YFP mice, resulting in a net increase in MU size in females but not in males. This result is consistent with previous findings on botulinum toxin-induced axonal sprouting (Moloney et al., 2017), where fast-fatigable motor axons were more likely to sprout in female mice. Interestingly, this increased dynamism is associated with an earlier onset of motor deficits and an exacerbated pathology in our female mice, as shown by the loss of grip strength and the neuromuscular and spinal cord histopathology. However, disease progression as measured by weight loss was equivalent in both sexes, consistent with previous reports in loxSOD1^G37R^ mice (Lobsiger et al., 2013; Mesci et al., 2015). These results suggest that excessive MU expansions may be maladaptive and detrimental to motor neuron survival in the end.

Sex-specific differences have been identified in other SOD1 mouse models and in patients carrying SOD1 mutations, but they did not consistently point toward a faster or more severe disease in one sex (Hayworth and Gonzalez-Lima, 2009; Kim et al., 2012; Zwiegers et al., 2014; Pfohl et al., 2015; Tang et al., 2019). For instance, male SOD1^G93A^ mice have an earlier onset than female SOD1^G93A^ mice (Hayworth and Gonzalez-Lima, 2009; Kim et al., 2012; Pfohl et al., 2015). Importantly, however, these differences depend on the presence of genetic modifiers (Heiman-Patterson et al., 2005). Furthermore, female mice from another SOD1^G37R^ line (line 29) have reduced survival and tend to have a delayed onset (Zwiegers et al., 2014), which is reminiscent of our present finding in loxSOD1^G37R^ mice. These results contrast with a recent study on Chinese familial ALS patients carrying SOD1 mutations which identified that women tended to have a slower progression and longer survival after onset (Tang et al., 2019). Of note, however, a large proportion of male ALS patients in the Chinese population are smokers (44.7% vs 1.7%; Chen et al., 2015) which has been previously associated with a substantial decrease (approximately one year) in survival (de Jong et al., 2012). Hence, sex seems to affect SOD1-mediated ALS onset and progression via a complex and unclear interplay with several other factors, including the specific disease mutation, the genetic background and environmental risk factors.

A number of factors could explain our observations. One interesting possibility is that excessive MU expansions are detrimental to motor neuron survival. A previous study on dopaminergic neurons identified that large axonal arborization size is associated with increased energetic load and enhanced vulnerability in Parkinson’s disease (Pacelli et al., 2015). Similarly, axonal length and complexity are predictive of NMJ vulnerability in ALS at the single motor neuron level (Tallon et al., 2016; Martineau et al., 2018). Hence, one could hypothesize that the excessive MU expansions in female SOD1^G37R^ mice could increase their energetic load during disease progression, exacerbating their stress. This possibility is consistent with the theoretical work of Le Masson et al.,(2014) showing that local energetic imbalance in distal axonal branches in ALS could propagate and give rise to global energetic failures, contributing to global neuronal degeneration. According to their simulations, large motor axons had higher ATP consumption and were much more susceptible to this energetic imbalance than small motor axons (Le Masson et al., 2014). Interestingly, previous studies identified sex-specific differences in mitochondrial function in SOD1^G93A^ mice that have been suggested to underlie female resilience in this line. Notably, female SOD1^G93A^ mice have increased activation of the mitochondrial unfolded protein response (UPRmt; Riar et al., 2017), reduced mitochondrial calcium accumulation (Kim et al., 2012) and improved preservation of Complex I function (Cacabelos et al., 2016). However, whether these sex-specific differences in mitochondrial function are mutation- or genetic-background-dependent remains to be determined. Nevertheless, together with the potential expansion-induced increase in bioenergetic load, these results suggest that alterations in mitochondrial function could contribute to the sex-linked phenotypic differences observed in ALS animal models.

Alternatively, increased levels of neuronal loss in female SOD1^G37R^ mice, as observed at P360, could in turn trigger more compensatory expansions from surviving MUs. This scenario could also explain why females tended to have higher global levels of NMJ denervation despite the similar amount of asynchronous NMJ losses between males and females. However, this scenario is not entirely consistent with the notion that female fast-fatigable motor axons are intrinsically more inclined to sprout (Moloney et al., 2017). Hence, additional studies aimed at manipulating MUs expansions levels in males and females are required to determine whether they represent an adaptive or a maladaptive response in female SOD1^G37R^ mice.

An important question is whether these findings on SOD1-mediated ALS are also applicable to other genetic forms of ALS or to sporadic ALS. Sex-specific differences have been observed in some non-SOD1 ALS mouse models and in the general ALS patient population, but they are as discordant as those observed in SOD1-mediated ALS. For instance, disease phenotype is more penetrant and rapidly progressing in females in one transgenic line of *C9orf72* mice (Liu et al., 2016). However, studies on *C9orf72* patient cohorts report conflicting results, with women having either a better (Rooney et al., 2017; Trojsi et al., 2019) or a worse (Watanabe et al., 2015) survival time after onset. Likewise, the incidence of sporadic ALS, but not familial ALS, is higher in men than in women, but bulbar-onset ALS is more common in women and is associated with a poor prognosis (McCombe and Henderson, 2010; Kiernan et al., 2011). Thus, as observed for SOD1-mediated ALS, sex seems to interact with several genetic and clinical factors that shape disease progression, such as the site of onset and the disease mutation. Whether sex-specific differences in MU dynamics also occur in non-SOD1-mediated forms of ALS and contribute to this phenotypic heterogeneity would be an important aspect to evaluate.

Altogether, we identified here a novel factor to consider in our understanding of the conundrum that represents sex-specific differences in ALS progression. These findings highlight the need to clarify the prevalence of sex-specific difference by performing additional detailed analyses across animal models of the disease. Furthermore, future large-scale studies looking at the influence of sex on disease onset, progression and motor function in gene-specific ALS patient cohorts could shed light on abstruse animal data and further our understanding of ALS pathogenesis.

## Synthesis

Reviewing Editor: Alfonso Araque, University of Minnesota Decisions are customarily a result of the Reviewing Editor and the peer reviewers coming together and discussing their recommendations until a consensus is reached. When revisions are invited, a fact-based synthesis statement explaining their decision and outlining what is needed to prepare a revision will be listed below. The following reviewer(s) agreed to reveal their identity: Matthew Butchbach, Paola Fabbrizio.

Both reviewers have found merit and interest on the manuscript and the results reported.

There are, however, some minor comments that need to be addressed. Addressing these concerns will enhance the strength of the manuscript.

I am looking forward to receiving a revised version of the manuscript.

Best Regards

Alfonso Araque

Specific comments of the reviewers:

Reviewer 1.

This study shows sex-related differences in neuromuscular junction denervation and axonal resprouting in a mouse model for a form of familial amyotrophic lateral sclerosis (ALS). These observations are important for the understanding of differences in disease progression observed between male and female ALS patients.

Specific comments

This report is a secondary analysis of previously published motor unit remodeling data in a mouse model for amyotrophic lateral sclerosis. Overall, the data are sound and the characterization of sex-related differences in neuromuscular junction denervation in ALS. The only concern raised during the evaluation of this manuscript is the applicability of these findings to ALS in general. Given that ALS has both sporadic and familial forms and that familial ALS is genetically heterogeneous, the authors should discuss if these sex-specific differences in motor unit remodeling are applicable to all forms of ALS or to just those resulting from mutations in SOD1.

Reviewer 2.

The authors carried out a study of the neuromuscular junctions in SOD1g37R mice and investigated the difference according to gender. This work is a deepening of data published in 2018 by the authors themselves. The article could give important and additional information to the current knowledge on the peripheral and central system of sod mice. In particular, bringing new evidence of the gender difference, always a debated topic in the field.

Specific comments

The article would benefit of being independent from the previous work. In the text there are too many references to the previous work that make it difficult to understand, without the knowledge of the previous one.

In “materials and methods” (line 85) the abbreviation MU should be made explicit and explained (as already reported in the previous work)

In “results” it would be useful to standardize the word male / female or with symbols or letters.

## Author Response

We would like to thank the reviewers and editors for their constructive suggestions to improve the manuscript and their positive assessment of our work. Please find enclosed the revised version of our manuscript entitled “Sex-specific differences in motor unit remodeling in a mouse model of ALS” (eN-TNWR-0388-19R1). We incorporated all the comments made by the reviewers. Modifications appear in red font in the main text. Please also find below a point-by-point rebuttal to their comments.We are eagerly looking forward for your reply.

Reviewer 1

Specific comments:

Comment 1: The only concern raised during the evaluation of this manuscript is the applicability of these findings to ALS in general. Given that ALS has both sporadic and familial forms and that familial ALS is genetically heterogeneous, the authors should discuss if these sex-specific differences in motor unit remodeling are applicable to all forms of ALS or to just those resulting from mutations in SOD1.

Reply: We thank the reviewer for this comment. Considering that sex seems to interact with several other genetic and clinical factors and that little is known about MU remodeling in these other forms of the disease, it is difficult to say whether sex-specific differences in motor unit remodeling also occur in these forms of the disease. Nevertheless, we added a new paragraph in the discussion (page 13) focusing on sex-specific differences in non-SOD1 mediated forms of ALS to highlight these conflicting results and this phenotypic heterogeneity. We argue that sex-specific difference in MU remodeling would be an important factor to investigate to understand this heterogeneity.

Reviewer 2.

Specific comments

Comment 1: The article would benefit of being independent from the previous work. In the text there are too many references to the previous work that make it difficult to understand, without the knowledge of the previous one.

Reply: We thank the reviewer for identifying this issue. We addressed it in several ways:

1) We added more details in the “In vivo imaging” section of the “materials and methods” (page 4 and 5) so that the manipulations are more easily understood without referring to the previous study.

2) We added two paragraphs at the beginning of the “Results” section (page 8). The first one summarizes the main finding of the previous study upon which we expand on and defines the terminology. The second one describes how the dataset was obtained and provides the sample size for each group (number of animals, motor units and NMJs). We believe this will provide the reader a clearer idea of the previous findings and how the data were obtained.

Of note, we were hesitant to present the in vivo imaging data in the first part of the results section without referring to the previous study. We want to make it clear to the reader that the comparisons and results presented in this section originate from a secondary analysis of the previously published dataset and not from new in vivo experiments. This is why we use terminology such as “the previously published dataset” or “secondary analysis”, even though it makes it a little more cumbersome for the reader.

We hope that the added details will alleviate this issue.

Comment 2: In “materials and methods” (line 85) the abbreviation MU should be made explicit and explained (as already reported in the previous work) Reply: The abbreviation MU is now defined at the beginning of the introduction (line 29).

Comment 3: In “results” it would be useful to standardize the word male / female or with symbols or letters.

Reply: Thank you for bringing this to our attention. We now exclusively use the words “male/female” in the text and in the parentheses.
